# Invasive Paget’s Disease of the Breast: Rash or Recurrence?

**DOI:** 10.7759/cureus.19216

**Published:** 2021-11-02

**Authors:** Austin J Pourmoussa, Starr K Mautner, Niloofar Nasseri-Nik, Katharine Lampen-Sachar

**Affiliations:** 1 Radiology, Baptist Health South Florida, Miami, USA; 2 Surgery, Baptist Health South Florida, Miami, USA; 3 Pathology, Baptist Health South Florida, Miami, USA; 4 Breast Imaging, Baptist Health South Florida, Miami, USA

**Keywords:** pruritic rash, atypical presentation, normal imaging, dermal invasion, paget's disease, breast cancer

## Abstract

Paget’s disease of the breast is rare among breast cancers. Secondary Paget’s disease of the breast presenting as local recurrence is even rarer, with limited published information on the overall prevalence. In this report, we present a case of secondary Paget’s disease of the breast presenting as a pruritic rash and skin changes with normal imaging. This patient’s case was unique as her presentation involved invasive Paget’s disease of the breast presenting as a local recurrence, with a diffuse rash covering the entirety of the right breast including the nipple-areolar complex, pathology examination showing dermal invasion, and a 20-year time interval between her initial treatment and presentation at our institution. Furthermore, diagnostic mammogram and breast MRI revealed no underlying suspicious findings within the breast tissue. In this case, the patient benefitted from mastectomy with removal of the affected skin, resulting in a clear margin and clinically favorable outcome. The patient did well postoperatively, did not receive any systemic adjuvant treatment, and is now under surveillance. Currently, there is insufficient data on the incidence of diffuse Paget’s disease of the breast with dermal invasion. It is important to recognize this atypical presentation to ensure timely diagnosis and treatment of affected patients.

## Introduction

Paget’s disease of the breast is rare among breast cancers, comprising approximately 1-3% of all breast tumors [1]. Mammary Paget’s disease was first described in 1874 by the English surgeon Sir James Paget [2] and is an adenocarcinoma characterized by histopathologic infiltration of neoplastic cells with glandular features in the epidermal layer of the nipple-areolar complex [3]. The clinical presentation is often initially mistaken as a benign dermatologic condition such as eczema or dermatitis of the nipple [4]. While primary Paget’s disease of the breast is rare, secondary Paget’s disease of the breast presenting as local recurrence is even rarer.

To our knowledge, there are no published statistics on the overall prevalence of secondary Paget’s disease of the breast presenting as local recurrence. Lohsiriwat et al. published on 861 breast cancer patients who underwent nipple-sparing mastectomy and electron beam intraoperative therapy with a median follow-up time of 50 months. Of the 36 patients in this study with local recurrences, seven recurred as Paget’s disease (0.8%) [3]. A larger retrospective review of 2,181 women with early-stage breast cancer treated with breast conservation revealed 183 patients with local recurrences, four of which were cases of Paget’s disease (2.2%) [5]. In this report, we present a case of secondary Paget’s disease of the breast presenting as extensive skin changes with normal imaging.

## Case presentation

**Figure 1:**
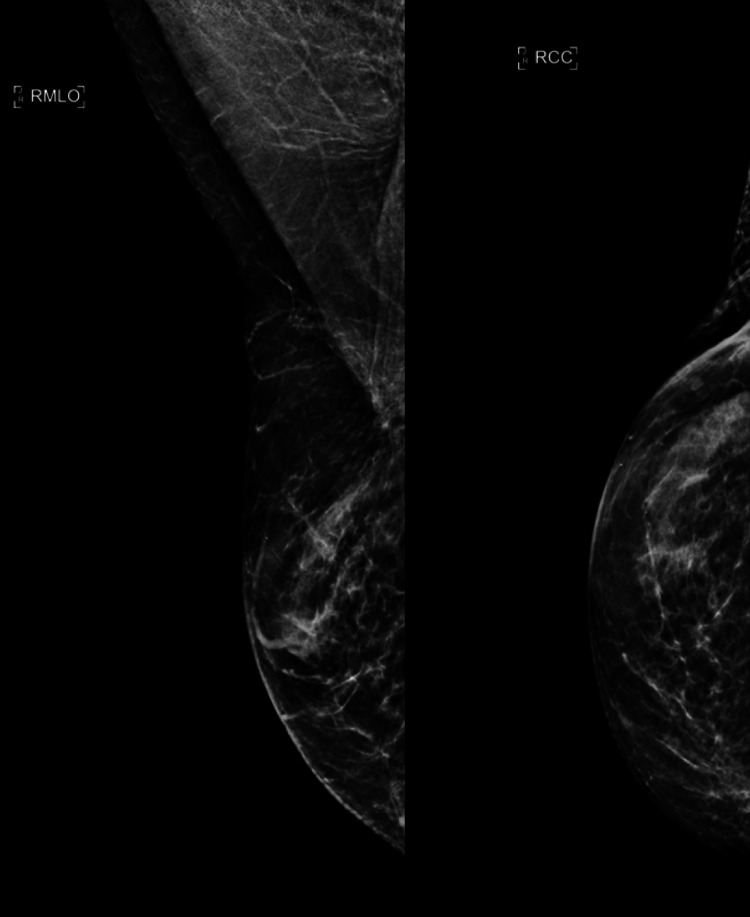
Right MLO (left image) and Right CC (right image) synthesized 2D reconstruction (C-View) demonstrating heterogeneously dense breasts with stable postsurgical changes in the right breast upper outer quadrant status post lumpectomy. Of note, no suspicious mass, groups of microcalcification, or skin thickening could be appreciated. MLO: mediolateral oblique view; CC: craniocaudal view

A 69-year-old female with a history of right-sided breast cancer in 1991 (reportedly Stage I, but details of her pathology at that time are unavailable) was treated at an outside institution with lumpectomy and axillary lymph node dissection at the time of her initial diagnosis. She received adjuvant treatment with chemotherapy, followed by whole breast radiation therapy, and took two years of tamoxifen, which was discontinued due to uterine polyps. In January 2021, she presented to our institution with extensive changes to the skin of her right breast and a recent skin punch biopsy of the right breast showing Paget’s disease with a focus suspicious for microinvasion. The patient reported an intermittent rash to her right breast for two and a half years with an occasional stinging sensation but without regions of palpable concern bilaterally. She reported the rash to be pruritic and scaly and with intermitted bleeding. A diagnostic mammogram performed at an outside institution showed no significant interval mammographic change over multiple years (Figure 1).

**Figure 2:**
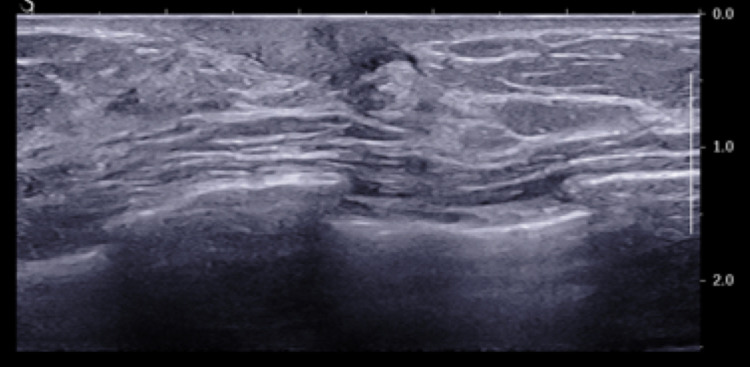
Representative static ultrasound image of the right subareolar breast demonstrating no suspicious mass.

**Figure 3:**
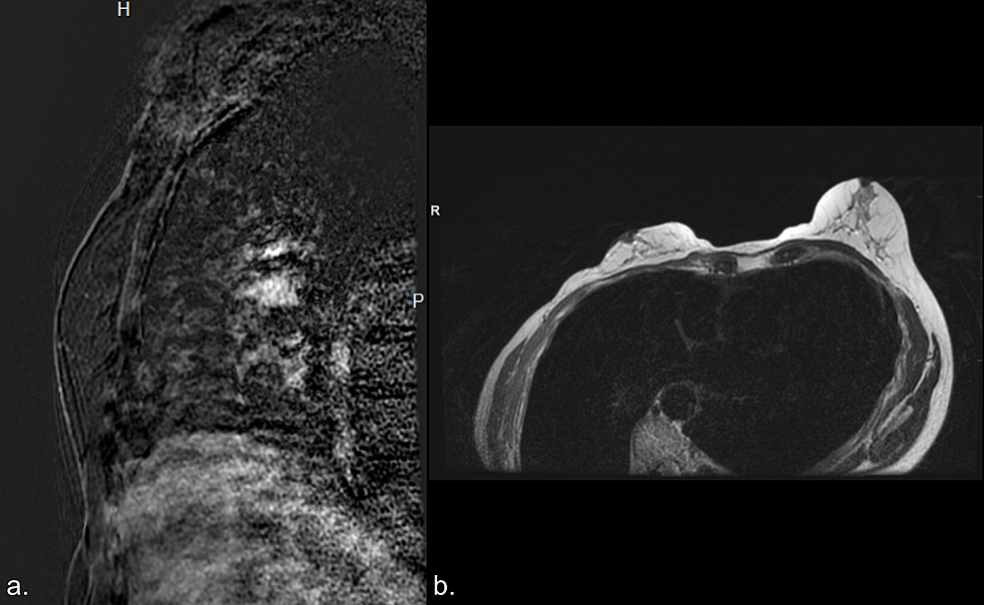
a. Post contrast T1 fat-suppressed subtracted sagittal image of the right central breast demonstrating no suspicious mass or non-mass enhancement in the right breast, including in the right subareolar breast. No suspicious skin thickening or enhancement was demonstrated. b. Axial T2 image of the breasts at the level of the nipple-areolar complex demonstrating asymmetric size of the right breast with respect to the left breast status post right breast lumpectomy. No skin thickening was demonstrated. H: head; P: posterior; R: right

No suspicious radiologic findings were present in the right breast to suggest disease underlying the skin. Static ultrasound imaging of the right subareolar breast demonstrated no suspicious mass (Figure 2). Breast MRI performed at an outside institution was of suboptimal quality, but demonstrated no gross suspicious findings with minimal background parenchymal enhancement (Figure 3). Her past medical history was significant for hypertension, osteoporosis, and hyperparathyroidism status post parathyroidectomy. The patient had a family history significant for liver cancer in her paternal uncle and grandmother but no reported family history of breast or ovarian cancer. Patient is of Ashkenazi Jewish ancestry but elected not to undergo genetic testing.

**Figure 4:**
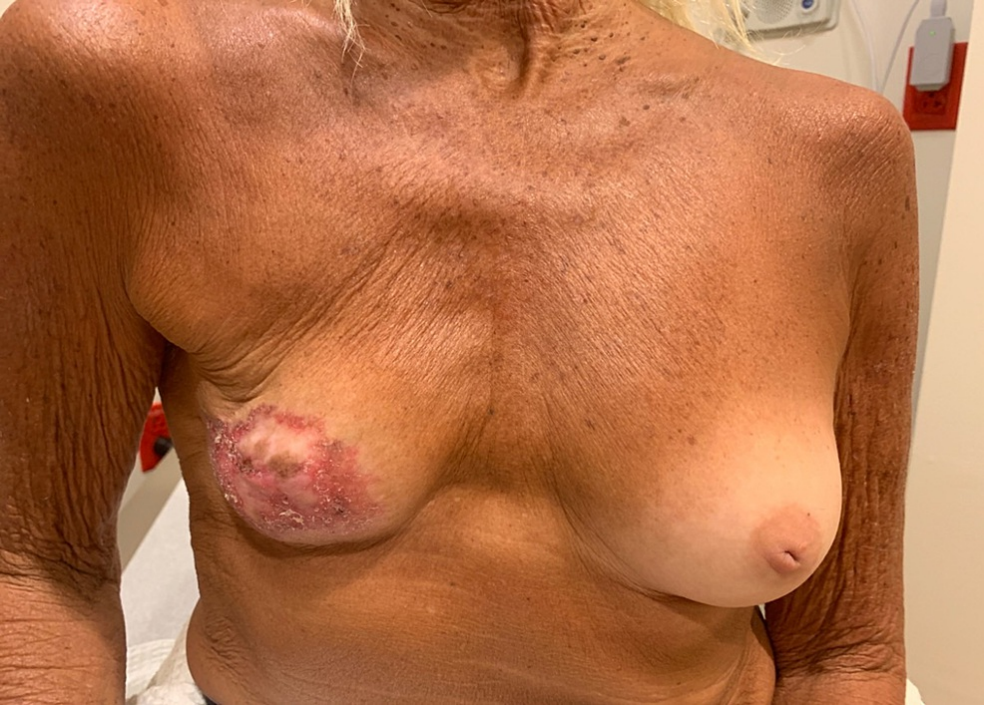
Clinical presentation showing right breast nipple-areolar complex obscured by skin changes.

On presentation to the surgical oncologist, the review of systems was positive for tenderness in the right breast and was negative for hematologic or lymphatic complaints. Clinical breast examination showed that the right breast was slightly smaller in size than her left breast, status post lumpectomy. There was an extensive area of right breast skin excoriation and erythema spanning 9 x 5.5 cm, which encompassed a majority of the skin of the breast. The right breast nipple-areolar complex was obscured by the skin changes (Figure 4). There were no dominant masses in either breast, no nipple discharge, and no palpable axillary lymphadenopathy bilaterally. 

Skin punch biopsy performed at an outside institution and reviewed by our pathology department showed two separate areas of the skin consistent with apocrine cancer/Paget’s disease and a focus suspicious for microinvasion. Given the extent of skin involvement, the surgeon recommended right breast mastectomy without immediate reconstruction to remove all of the affected skin. Given that the patient had prior complete axillary lymph node dissection, a negative clinical axillary exam, and negative imaging, additional staging of the axilla was not performed to limit her risk of lymphedema.

**Figure 5:**
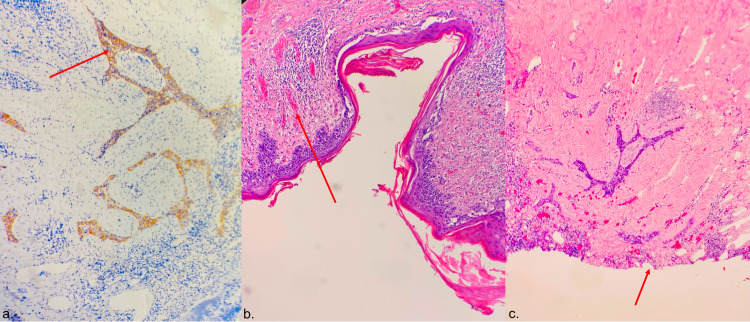
a. HER2 positive nests of invasive Paget’s disease (arrow); b. Neoplastic cells in the epidermis (arrow); c. Invasive Paget’s disease, epidermis was ulcerated and sloughed off at this focus (arrow). HER2: human epidermal growth factor receptor 2

Final pathology of the right breast mastectomy specimen showed irregular nests of poorly differentiated tumor cells infiltrating the superficial dermis in the background with epidermal Paget disease and dermal scar (Figure 5). Paget cells and invasive nests were positive for cytokeratin 7 (CK7) with equivocal staining for human epidermal growth factor receptor 2 (HER2) (2+/3). The diagnosis of invasion was confirmed by negative staining for myoepithelial cells on p63/AE1/3, calponin, and smooth muscle myosin heavy chain (SMMHC)/AE1/3 and p40. As no primary breast tumor (in-situ or invasive mammary carcinoma) was seen in the underlying breast and due to the presence of invasive Paget nests in the dermis immediately underneath the epidermal Paget, the tumor was classified as “invasive Paget disease.” Final surgical pathology revealed free margins.

On follow-up, the patient healed well and had no specific complaints. The patient was seen by medical oncology to discuss the role of adjuvant anti-HER2 directed therapy, and she decided not to pursue any additional treatment. The patient remains clinically free of disease at six months following surgery.

## Discussion

Paget’s disease of the breast predominately affects the nipple-areolar complex and typically presents with erythema, pruritis, flaking, and/or crusting of the nipple with or without a flattened nipple and discharge. Based on published literature, approximately 0.8-2.2% of patients with local recurrence following definitive treatment of breast cancer present with invasive Paget’s disease [3,5]. The study by Lohsiriwat et al. [3] found a 0.8% local recurrence rate of Paget’s disease in patients who received electron beam intraoperative radiotherapy. This is a unique patient population and is in contrast to our patient who received adjuvant whole breast radiation therapy. The timing of presentation is also worth noting, as the study by Lohsiriwat et al. had a median follow-up time of 50 months while our patient presented 20 years after her initial treatment. 

On pathology examination, Paget’s disease is characterized by invasion of the epidermis by Paget cells, a type of malignant glandular epithelial cell [6]. The incidence of Paget’s disease of the breast invading the dermis is rare and not well described in the literature [7]. Furthermore, the incidence of Paget’s disease presenting as a diffuse rash covering the entire breast is also rare and not well described in the literature.

Our patient’s case was unique as her presentation involved invasive Paget’s disease of the breast presenting as a local recurrence with a diffuse rash covering the entirety of the right breast including the nipple-areolar complex, pathology examination showing invasion of the dermis, and a 20-year time interval between her initial breast cancer treatment and presentation at our institution. Furthermore, her breast imaging was unremarkable. In this case, the patient benefitted from mastectomy with removal of the affected skin, resulting in a clear margin and clinically favorable outcome. The patient ultimately did well after her treatment and has maintained close follow-up.

Although this patient's presentation was unusual, a collaborative effort between the departments of Surgery, Pathology, and Radiology yielded a positive outcome. It is important to identify patients at risk in order to facilitate the appropriate treatment. Teamwork between specialties is an important factor to optimize timely diagnosis and treatment, ultimately maximizing benefit to the patient. In a patient with a past history of breast cancer, new skin changes should not be ignored and the treating clinician should consider timely skin punch biopsy in order to rule out recurrence.

## Conclusions

Although rare, invasive Paget’s disease of the breast can present as a local recurrence of primary breast cancer with normal radiologic findings. In rare cases, Paget’s disease of the breast may present diffusely across the entirety of the breast, and may also invade the dermis. There is insufficient data on the incidence of diffuse Paget’s disease of the breast with dermal invasion. This case represents a unique patient with the full constellation of rare events, including invasive Paget’s disease of the breast presenting as a local recurrence affecting the entirety of the breast extending beyond the nipple-areolar complex, with the pathology report showing dermal invasion and normal radiologic findings. Given the limited pathology information on the patient's initial diagnosis from an outside institution and remote time interval, it is possible that her case represented a new primary Paget's disease of the breast as opposed to a secondary recurrence. At any rate, it is important to recognize this atypical presentation and consider skin punch biopsy in order to ensure timely diagnosis and treatment of affected patients.
